# Subsequent Physical Activity–Related Musculoskeletal Injuries in University Students: The Role of Body Composition, Training Weekly Load, and Physical Activity Intensity

**DOI:** 10.3390/jcm15030961

**Published:** 2026-01-25

**Authors:** Edyta Kopacka, Jarosław Domaradzki

**Affiliations:** 1 Doctoral School, Wroclaw University of Health and Sport Sciences, 51-612 Wrocław, Poland; edyta.kopacka@awf.wroc.pl; 2 Department of Biological Principles of Physical Activity, Wroclaw University of Health and Sport Sciences, 51-612 Wrocław, Poland

**Keywords:** subsequent injury, SIC-2.0, training load, students, body composition, sport type

## Abstract

**Background/Objectives**: Subsequent musculoskeletal injuries are frequent among physically active young adults, yet the roles of body composition, training weekly load (TWL), and physical activity intensity in subsequent injury occurrence remain unclear. This study examined the associations of body composition indices and training-related variables with subsequent injuries in university students and explored whether combining key markers from body composition and training exposure improves discrimination compared with single markers. **Methods**: The analysis included 418 students from two cohorts merged after confirming negligible between-cohort differences. Participants completed questionnaires on injury history and physical activity and underwent standardized anthropometric and body composition assessments. Intrinsic factors included fat mass index (FMI) and skeletal muscle mass index (SMI), while extrinsic factors comprised training weekly load (TWL), total physical activity (TPA), and vigorous activity percentage (VPA%). Subsequent injury (yes/no) served as the primary outcome. Injuries were assessed retrospectively over the preceding 12 months; subsequent injury was defined as ≥1 injury occurring after a previous (index) injury within this recall period. Analyses used univariate and multivariable logistic regression and exploratory Receiver Operating Characteristic (ROC) analyses for individual markers and combined models. **Results**: SMI was associated with subsequent injury (OR = 1.09, 95% CI: 1.03–1.15). TWL showed a weak, non-significant association (OR = 1.03, *p* = 0.307). Models combining SMI and TWL, including their interaction, did not meaningfully improve discrimination compared with SMI alone. ROC analyses indicated limited discriminatory ability across models (AUCs < 0.65), suggesting poor accuracy for identifying individuals with subsequent injury based on these markers. **Conclusions**: The examined body composition, training weekly load (TWL), and physical activity measures alone or combined showed limited discriminatory utility for subsequent injury status in this cross-sectional sample. These findings support the multifactorial nature of injury susceptibility and indicate that simple anthropometric or TWL-based measures are not suitable as standalone screening tools for subsequent injury in active university populations.

## 1. Introduction

Musculoskeletal injuries represent a frequent health burden among physically active individuals, particularly young adults engaged in recreational and competitive activities. In a large collegiate survey, the 1-year prevalence of sports injuries reached 50.01% among Japanese university athletes [1]. Recent biomechanical evidence also suggests that optimizing landing strategies may help reduce lower-limb injury risk [2]. Beyond first-time injuries, subsequent injuries represent an important clinical and practical problem because they may disrupt training continuity, reduce physical performance, and increase longer-term health consequences [3]. Subsequent injuries may occur due to incomplete recovery, persistent functional deficits, or an inappropriate return to physical activity, and they can be categorized according to whether they affect the same site and tissue as the index injury [4,5]. Understanding factors associated with subsequent injuries is therefore essential for informing prevention strategies and supporting safe participation in physical activity.

Previous research has identified several determinants of musculoskeletal injuries, including individual characteristics and body composition, as well as prior injury history [6]. Evidence syntheses indicate that hamstring strain injuries have multifactorial determinants, with neuromuscular and exposure-related factors often discussed as relevant contributors, although effects are typically modest and context-dependent [7]. In addition, systematic evidence indicates that training exposure and workload are associated with injury outcomes in physically active populations, although the strength of these associations varies across settings and outcome definitions [8,9]. Training exposure and load (e.g., training weekly load) may contribute to injury occurrence, particularly when increases in training dose are rapid and recovery is insufficient, potentially exceeding tissue capacity [8,9]. Despite these advances, most studies have focused primarily on initial injuries rather than subsequent injuries, and the determinants of repeated injury events remain less well understood in student populations [7].

Analyzing subsequent injuries is crucial because they may involve different body sites or tissue types than the initial event, indicating that previously injured individuals can be vulnerable to broader injury patterns [10]. Importantly, the risk of a subsequent time-loss injury appears to be highest immediately after return to play; for example, [10] reported a 9.4% injury risk in the week of return to play compared with a 3.6% baseline risk. Moreover, re-injuries may occur shortly after an initial injury and during return-to-play, when unresolved deficits combined with ongoing training exposure can increase vulnerability to a further episode [11]. To better interpret these patterns, classification approaches allow researchers to distinguish between recurrent, related, and unrelated subsequent injuries and to examine injury trajectories more precisely [12]. Such approaches improve the interpretability of repeated injury events and support the development of targeted prevention strategies [13].

Despite the growing body of evidence on musculoskeletal injuries in young adults, university students remain understudied with respect to subsequent injury occurrence. In particular, there is a lack of studies that jointly examine the interplay between key intrinsic body-composition indices reflecting adiposity and muscularity, specifically fat mass index (FMI) and skeletal muscle index (SMI) and a key extrinsic exposure metric, training weekly load (TWL), in relation to subsequent injuries in this population [14]. Although body composition indices have been associated with injury occurrence in physically active individuals [15], most prior work in student populations has focused on first-time injuries or general musculoskeletal symptoms rather than on subsequent injuries following an initial episode. Given that musculoskeletal injuries are common among university students and may impair daily functioning and academic performance [16], clarifying how FMI and SMI relate to subsequent injury outcomes in the context of TWL is important for developing evidence-informed prevention strategies. Furthermore, applying extended classification frameworks for subsequent injuries can provide deeper insight into mechanisms leading to repeated injury events and help differentiate recurrent, related, and unrelated outcomes [17]. Together, these gaps form the rationale for the present study.

This study is the first to comprehensively analyze subsequent musculoskeletal injuries among university students by focusing on the joint contribution of body composition indices (FMI and SMI) and training weekly load (TWL). Using an established subsequent injury classification framework, this study differentiates between recurrent, related, and unrelated injuries to characterize injury trajectories more precisely [17]. Accordingly, this study addresses the following research questions:

(1) What are the independent and combined associations of FMI, SMI, and TWL with the occurrence of subsequent musculoskeletal injuries (recurrent, related, or unrelated) in university students?
(2) To what extent do FMI, SMI, and TWL contribute to explaining differences in subsequent injury outcomes, and what are the main explanatory limitations of these associations within the present study design?

We hypothesized that FMI, SMI, and TWL would each be associated with subsequent injury occurrence, and that considering body composition, training weekly load (TWL), and physical activity measures jointly would provide a more comprehensive explanatory perspective than single-domain analyses, without implying predictive performance.

## 2. Materials and Methods

The present analysis is based on data from two independently recruited cohorts of physically active university students assessed between 2022 and 2023 using an identical measurement protocol. The cohorts were merged to increase statistical power and improve the robustness of injury-related analyses, particularly for subsequent and recurrent injury outcomes characterized by relatively low event counts. For the purposes of this study, only participants with complete data for all variables relevant to the planned analyses were included, which explains differences between the current sample size and those reported previously.

Prior to merging, the cohorts were compared across all variables included in the present analyses; standardized mean differences below 0.10 supported their treatment as a single study population.

### 2.1. Study Design

The investigation followed a cross-sectional framework, and participants were enrolled from a university student population using a non-probability (convenience) sampling strategy. A cross-sectional design was adopted because injury history, including prior and subsequent injuries, was assessed retrospectively within a defined recall period using a standardized questionnaire. Data collection was carried out at the Wroclaw University of Health and Sport Sciences during 2022–2023, involving students from physical education, sport, and physiotherapy degree programs.

Data from two independently recruited cohorts were combined to increase statistical power and ensure stable estimation of associations between subsequent musculoskeletal injury occurrence and intrinsic and extrinsic risk factors. Prior to merging, cohort comparability was verified as described above.

Participants completed an online questionnaire assessing musculoskeletal injury occurrence, physical activity patterns, and training characteristics, followed by an in-person laboratory assessment including anthropometric and body composition measurements.

### 2.2. Ethics

Ethical clearance for the study was granted by the Senate Research Ethics Committee of the Wroclaw University of Health and Sport Sciences (approval no. 13/2022). All stages of the research were carried out in line with the ethical principles defined by the Declaration of Helsinki. Before enrolling in the project, students received comprehensive information regarding the purpose of the study, the assessment procedures, and the conditions under which their data would be stored and used. Each participant provided electronic informed consent prior to the commencement of data collection.

### 2.3. Sample Size

The planned sample size was guided by methodological recommendations for multivariable analyses in epidemiological research. For logistic regression, we followed the widely used heuristic method of approximately the number of outcome events per model parameter as a practical guideline to support reliable model estimation, including interaction terms [18,19].

Alongside this rule-of-thumb, we also performed an a priori sample-size calculation based on the conventional proportion formula, assuming a 95% confidence level and a prespecified margin of error (δ) [18,19]:

$$n=(\frac{1.96}{\delta }{)}^{2}\times p\left(1-p\right).$$

With δ set to 0.05 and p fixed at 0.5 (worst-case variance), the resulting minimum sample size was 385. To account for potential missingness and incomplete records, we inflated this estimate by 20%, yielding a target sample size of approximately 460 participants. These parameters were chosen to provide sufficient power for sex-stratified analyses. In subgroup models where the “10 events per predictor” guideline could not be fully met, this criterion was treated as a flexible recommendation.

### 2.4. Participants

In total, 454 university students participated (219 men, 48%; 235 women, 52%). Participants were drawn from students studying Physical Education, Sport, and Physiotherapy at the Wroclaw University of Health and Sport Sciences during the 2022–2023 academic year. Invitations to join the study were extended during scheduled classes, with no obligation to participate. The proportion of men and women in the final sample closely mirrored the typical sex distribution within these academic programs.

Initial eligibility criteria specified that participants had to be physically active students who regularly attended on-site university classes. To keep the sample comparable in terms of habitual physical activity exposure, we excluded students who participated in university-level competitive sport or were enrolled in elite performance programs. University-level competitive sports participation was defined as current membership in officially recognized university sports teams competing in inter-university or national-level competitions. Enrollment in elite performance programs referred to participation in structured, high-performance training pathways characterized by systematic coaching, competition schedules, and training volumes exceeding standard curricular requirements.

Eligibility for inclusion was assessed using a standardized self-report screening question administered during recruitment, in which participants declared whether they were currently involved in university sports teams or elite athletic programs. Individuals responding affirmatively were excluded from participation. Exclusion criteria were: (1) refusal to participate, (2) incomplete data for key study variables, (3) exemption from mandatory university physical classes lasting longer than two consecutive weeks, and (4) presence of an acute musculoskeletal injury within one month prior to the assessments.

Out of 454 participants, 36 were excluded due to missing data in key variables (questionnaires, including injury occurrence or measurements, mainly balance—not analyzed in this work), which were treated as block-missing cases.

A further 13 participants were missing data for only one variable; these missing values were addressed using multiple imputation, as detailed in the subsequent section. After applying data cleaning procedures and handling missing values through multiple imputation, the final analytical cohort comprised 418 students. The average total physical activity levels, expressed in MET·min/week, were above the IPAQ-defined high-activity criterion (≥3000 MET·min/week) in both men (3608 ± 1357) and women (3019 ± 1001).

### 2.5. Anthropometric Measurements

Anthropometric data collection took place at the Biokinetics Research Laboratory of the Central Research Laboratory, Wroclaw University of Health and Sport Sciences. The laboratory complies with a certified Quality Management System aligned with PN-EN ISO 9001:2015 (Certificate No. PW-15105-22X) [20].

Stature was recorded in duplicate to the nearest 0.1 cm using a GPM anthropometer (GPM Instruments GmBH, Susten, Switzerland). Assessment of body mass and body fat percentage was carried out by means of bioelectrical impedance analysis (BIA) using the InBody 230 device (InBody Co., Ltd., Cerritos, CA, USA). Participants underwent testing without footwear and in light sports attire, in accordance with the manufacturer’s guidelines. Body mass index (BMI) was then computed using the following equation:

$$\mathrm{BMI}=\frac{\mathrm{body}\;\mathrm{mass}\;\left[\mathrm{kg}\right]}{\mathrm{body}\;\mathrm{height}{\;[m}^{2}]}$$

Similarly, Fat Mass Index and Skeletal muscle Mass Index were calculated using formula:

$$\mathrm{FMI}=\frac{\mathrm{body}\;\mathrm{fat}\;\mathrm{mass}\;\left[\mathrm{kg}\right]}{\mathrm{body}\;\mathrm{height}{\;[m}^{2}]}$$

$$\mathrm{SMI}=\frac{\mathrm{body}\;\mathrm{skeletal}\;\mathrm{muscle}\;\mathrm{mass}\;\left[\mathrm{kg}\right]}{\mathrm{body}\;\mathrm{height}{\;[m}^{2}]}$$

In the present study, BMI was used solely as a descriptive variable. To avoid redundancy and potential collinearity, only body composition indices—fat mass index (FMI) and skeletal muscle index (SMI)—were included as independent variables in the analytical models.

### 2.6. Questionnaire Measurements

Injury Occurrence—Injury History Questionnaire (IHQ)

Musculoskeletal injury history from the last 12 months was evaluated with the use of the standardized Injury History Questionnaire (IHQ), a commonly used instrument in epidemiological studies of physically active individuals [14]. Participants reported the number and types of injuries related to sport, recreation, or everyday activities, including information on the injured body region and the duration of any resulting time-loss. For statistical analysis, responses were recoded into a binary variable reflecting the presence (1) or absence (0) of injury, which served as the main outcome measure. Previous studies in young adult and university student samples have demonstrated good test–retest reliability of the IHQ.

Subsequent injury classification

Based on the Injury History Questionnaire (IHQ), participants who reported at least one injury were additionally asked to indicate whether the injury occurred at the same anatomical location and involved the same injury type as any previously reported injury. Using this information, subsequent musculoskeletal injuries were classified into three mutually exclusive categories.

Recurrent injuries were defined as injuries occurring at the same anatomical location and of the same type as a previous injury. Related injuries were defined as injuries occurring at the same anatomical location but involving a different injury type, or injuries of the same type occurring at a different anatomical location. Unrelated injuries were defined as injuries occurring at a different anatomical location and involving a different injury type compared with any previously reported injury.

This classification was applied using predefined operational criteria derived from self-reported injury characteristics and was used to address study aims related to the differentiation of injury types.

For analytical purposes, subsequent injuries were further operationalized according to case numbers. While recurrent, related, and unrelated injuries were initially identified, injuries classified as related and unrelated were combined into a single category labeled “other subsequent injury” due to the limited number of cases in these subgroups. This approach ensured sufficient statistical power and stable estimation in the statistical analyses.

Physical Activity—International Physical Activity Questionnaire (IPAQ)

Levels of physical activity were measured using the Polish version of the International Physical Activity Questionnaire—Long Form (IPAQ-LF) [21]. The questionnaire was completed online through the Google Forms platform. The independent variables were: total physical activity—TPA [MET/min/week], and vigorous component (VPA) presented as a percentage of TPA {%}.

Training weekly load (TWL)

Training Weekly Load (TWL) was derived from self-report items included in the lifestyle questionnaire. Participants were asked: (1) “On average, how many structured training sessions do you perform per week?” and (2) “On average, how long does one training session last?” (reported in minutes). TWL was calculated as the product of weekly training frequency and average session duration, and expressed as minutes per week, providing an estimate of the total structured training volume (time-based exposure)accumulated across a week. This pragmatic approach was selected as it is suitable for cross-sectional, questionnaire-based studies in heterogeneous, non-elite student populations, where prospective session-level measures of internal load (e.g., session-RPE) are not feasible and may be affected by recall bias. This approach has been widely used in studies of physically active students and recreational athletes, offering a practical indicator of habitual training exposure.

### 2.7. Treatment of Incomplete Data and Imputation Strategy

Data incompleteness was observed for some questionnaires results (Questionnaire of Eating Behaviors—QEB and Pittsburgh Sleep Quality Index—PSQI) and balance assessments. The missing-data mechanism was evaluated using a logistic regression–based Missing Completely At Random (MCAR) test, in which PSQI missingness was regressed on all 16 QEB dietary items. The likelihood ratio test (χ^2^ = 18.64, df = 16, *p* = 0.288) indicated no dependence on observed, supporting the assumption that missingness followed a Missing Completely at Random (MCAR) pattern. Balance measures were excluded from the missingness diagnostics because they were not included in the primary analytical framework.

Missing values in multivariable analyses were managed by applying a multiple imputation approach based on chained equations (MICE; mice v3.14.0). Twenty imputed datasets were generated using predictive mean matching for continuous variables, logistic regression for binary variables, and polytomous regression for ordinal dietary items. All analyses were conducted in R (RStudio 2025.09.1+401), with convergence confirmed through standard diagnostic plots.

Although the missing values did not concern the variables included in the present injury-related analyses, the imputation procedure is reported because it affected the composition of the overall dataset. Multiple imputation allowed all participants with partially incomplete records to remain in the analytical pool, ensuring that the final dataset consisted of 418 individuals rather than a smaller, case-wise reduced sample. In other words, imputing missing values preserved the full sample structure used across the study, while maintaining appropriate statistical integrity for subsequent modeling.

### 2.8. Statistics

Data analyses were carried out with the use of Statistica 14.0 (TIBCO Software Inc., Palo Alto, CA, USA) as well as RStudio (v2025.09.1+401). Assumptions of normality and homoscedasticity for continuous variables were verified using the Shapiro-Wilk and Levene’s tests, respectively, before further modeling. Baseline characteristics are summarized using means ± standard deviations with 95% confidence intervals or frequencies and proportions, as appropriate.

**Figure 1:**
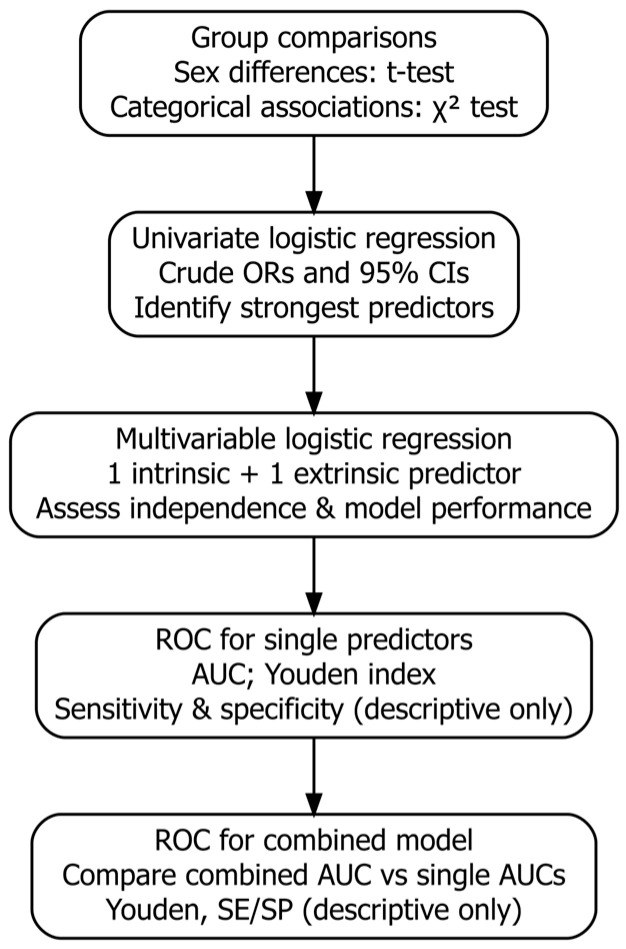
Analytical flowchart illustrating the sequential statistical procedures applied in the study, from data screening and descriptive analyses to regression modeling and receiver operating characteristic (ROC) analyses for single and combined predictors of subsequent injury.

The sequence of statistical analyses and decision steps applied in the study is summarized in an analytical flowchart (Figure 1), providing an overview of the methodological process from preliminary data screening to multivariable modeling and ROC-based evaluation.

Simple comparisons

Sex-related differences were assessed by applying independent t-tests for continuous variables and chi-square tests for categorical variables.

Univariate logistic regression

Following the exploratory stage, separate univariate logistic regression models were estimated for each intrinsic and extrinsic variable to assess their individual associations with subsequent injury (injured vs. non-injured). These models provided crude odds ratios (ORs) and 95% confidence intervals, allowing identification of the strongest single predictors within each risk-factor domain.

Multivariable logistic regression

Variables showing the strongest independent relationships in the univariate models (sex, FMI or SMI and TPA, VPA, TWL or EXP)—were then jointly entered into multivariable logistic regression models. This step allowed assessment of whether intrinsic and extrinsic predictors remained significant when controlling each other, and whether their combined effects improved model performance relative to single-factor models.

ROC analysis for single predictors

To quantify the discriminatory ability of the strongest body-composition and training/physical activity predictors, receiver operating characteristic (ROC) curves were generated for each variable separately. The discriminatory ability of the predictors was assessed by calculating the area under the curve (AUC). Optimal threshold values were established using the Youden index, with sensitivity and specificity reported for each variable. Cut-off points, sensitivities, and specificities were reported for descriptive and comparative purposes only and should not be interpreted as thresholds for practical screening or clinical decision-making.

ROC analysis for combined predictors

Finally, a ROC curve was constructed for the multivariable model including both the body-composition and training/physical activity predictors. Comparing this combined AUC to the AUCs of the two single-predictor models allowed evaluation of whether the cumulative effect of the strongest risk factors provided superior discrimination relative to individual predictors alone, and whether integrating intrinsic and extrinsic characteristics meaningfully enhanced injury-risk classification. Accordingly, sensitivity, specificity, and Youden index values are presented for descriptive and comparative purposes only and should not be interpreted as clinically actionable screening thresholds.

The significance threshold was set at *p* < 0.05. Interaction effects were visualized in R using ggplot2 (v. 4.0.1.).

Generative AI tools were used in accordance with COPE recommendations and MDPI transparency requirements and were limited to preparatory, editorial, and organizational support. No AI systems were involved in statistical analyses, interpretation of results, or formulation of scientific conclusions. Chat Academia (v1.0, 2025), Scholarcy (v4.0, 2025), NotebookLM (v1.3, 2025), and Elicit (v2.0, 2025) were used for literature exploration, preliminary summaries, and identification of common methodological approaches, with all outputs verified manually. ChatGPT (OpenAI, GPT-4.1, 2025) supported language editing, early drafting, and access to technical documentation for R (v. 4.5.2.) packages. All AI-assisted content was reviewed, edited, and approved by the authors, who take full responsibility for the final manuscript.

## 3. Results

### 3.1. Participant Characteristics

Baseline characteristics are summarized in Table 1. Data are reported as mean ± standard deviation with 95% confidence intervals. Anthropometric parameters differed significantly by sex, with males showing higher values across all somatic measures (all *p* < 0.05). In addition, males reported significantly higher physical activity levels than females (*p* < 0.001), as well as statistically significantly more experienced (more years of training) (*p* < 0.001). However, both sexes did not differ in vigorous physical activity (0.340), nor in training weekly load (*p* = 0.200).

Across the full sample (N = 418), slightly more than half of the students (51.4%) reported at least one injury during the previous 12 months. When comparing categories by sex, males were significantly more likely than females to report any injury (χ^2^ = 4.35, *p* = 0.037) (Table 2). No statistically significant sex differences were observed for subsequent injury (χ^2^ = 1.27, *p* = 0.259), recurrent injury (χ^2^ < 0.01, *p* = 0.998), or other subsequent injuries (χ^2^ = 1.18, *p* = 0.277).

Subsequent injuries accounted for approximately one-third of all injury cases (34.9%; n = 146). Because this category includes both recurrent injuries and other subsequent injuries, it is possible for a participant to have experienced both a repeated injury to the same anatomical site and additional new injuries within the same 12-month period. Thus, the category of “subsequent injury” captures the full spectrum of multiple injury patterns occurring after the initial event.

### 3.2. Single-Predictor Logistic Regression Analyses of Subsequent Injury

In the univariate logistic regression models, most intrinsic and extrinsic variables demonstrated weak or negligible associations with the likelihood of sustaining a subsequent injury. BMI was not included in regression models to avoid overlap with body composition indices. Neither sex, fat mass index (FMI), total physical activity (TPA), vigorous activity percentage (VPA%), nor training experience (EXPER) showed statistically meaningful effects, with odds ratios hovering around 1.00 and confidence intervals broadly overlapping the null value.

Among the examined variables, skeletal muscle mass index (SMI) emerged as the only significant predictor, with higher SMI associated with a modest increase in subsequent injury risk (OR = 1.09, 95% CI: 1.03–1.15, *p* = 0.002, Cohen’s d = 0.05) (Table 3). Training weekly load (TWL) showed a slightly stronger crude effect relative to other extrinsic factors (OR = 1.03, 95% CI: 0.98–1.08, Cohens’s d = 0.02), although this association did not reach statistical significance (*p* = 0.307) and its confidence interval suggested a weak and imprecise relationship.

Overall, the univariate analyses showed that individual risk factors had limited predictive relevance, and even the comparatively more influential variables—SMI and TWL—accounted for only a small fraction of the variability in subsequent injury incidence.

### 3.3. Joint and Interactive Effects of Body Composition and Training Variables on Subsequent Injury

In the multivariable logistic regression model including SMI, TWL, and their interaction, none of the predictors reached statistical significance. The main effects of SMI (OR = 1.07, 95% CI: 0.98–1.18, *p* = 0.139, Cohen’s d = 0.04) and TWL (OR = 0.98, 95% CI: 0.78–1.23, *p* = 0.833, Cohen’s d = 0.01) were small and imprecise, and the interaction term showed no evidence of synergistic or multiplicative effects (OR = 1.00, 95% CI: 0.99–1.02, *p* = 0.743), indicating a clearly non-significant interaction effect (Table 4). These findings indicate that neither higher skeletal muscle mass nor weekly training load, nor their combination, meaningfully increased or decreased the likelihood of sustaining a subsequent injury.

### 3.4. ROC Curve Analysis of Individual and Combined Predictors of Subsequent Injury

**Figure 2:**
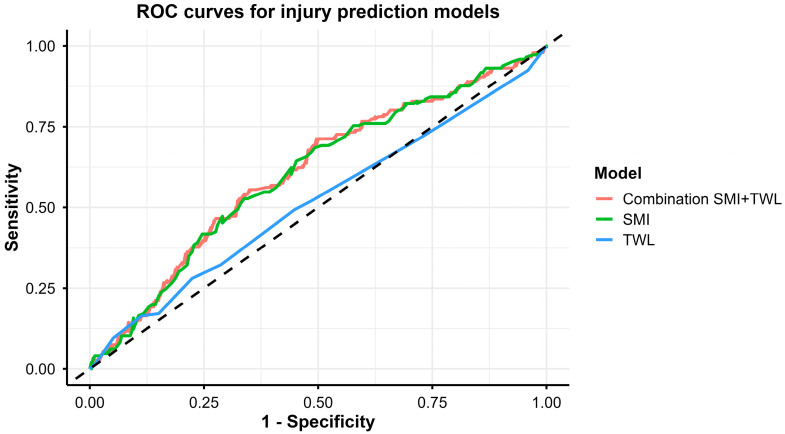
ROC curves were generated for the single markers—skeletal muscle mass index (SMI) and training weekly load (TWL) and for their combined model (SMI + TWL). The dashed line represents the line of no discrimination (AUC = 0.50).

These results indicate that FMI and AA were associated with injury probability in an additive manner, with no clear evidence of an interaction between the two factors. The ROC analysis evaluated the ability of SMI (body composition) and TWL (training exposure), alone and in combination, to discriminate students with subsequent injuries. As shown in Table 5 and Figure 2, all models exhibited only modest discriminatory performance, with AUC values around 0.60. The highest AUC was observed for the combined model including SMI and TWL (AUC = 0.607, 95%CI: 0.550–0.664, *p* < 0.001), slightly exceeding that of SMI alone (AUC = 0.604, 95%CI: 0.548–0.661, *p* < 0.001), whereas TWL alone showed near-chance performance (AUC = 0.516, 95%CI: 0.457–0.576, *p* = 0.596). Optimal cut-off points derived from the Youden index yielded sensitivities of 0.712 and 0.685 and specificities of 0.504 and 0.507 for the combined model and SMI alone, respectively, indicating limited trade-offs between true-positive and false-positive rates. AIC values were comparable for the combined model and SMI alone (536.4 and 534.7, respectively), while TWL alone yielded the poorest model fit (AIC = 543.9).

## 4. Discussion

This study examined how body composition indices, training weekly load (TWL), and physical activity measures relate to subsequent musculoskeletal injuries in physically active university students. Overall, the observed associations were weak. Univariate models showed only minimal associations between individual factors and subsequent injury, with SMI and, to a lesser degree, TWL emerging as the relatively strongest but still modest markers. The combined model indicated that neither SMI nor TWL, nor their interaction contributed meaningfully to subsequent injury status. Consistent with these findings, exploratory ROC analyses showed poor discrimination (AUC only marginally above ≈0.60 and <0.65 across models), indicating that practically these models are not useful for screening or individual-level stratification of subsequent injury in this population.

A key finding was that SMI was the only intrinsic/extrinsic variable significantly associated with subsequent injury; however, the magnitude of this association was minimal (OR ≈ 1.09) and corresponds to a trivial effect (Cohen’s d ≈ 0.05), which limits its explanatory relevance and practical value. This pattern is consistent with broader evidence suggesting that many modifiable morphological or neuromuscular characteristics show weak or inconsistent associations with injury outcomes and often provide limited standalone utility beyond non-modifiable factors such as age and previous injury [7]. Similarly, prospective research in professional football suggests that comprehensive strength-testing protocols may have limited value as screening tools for discriminating future musculoskeletal injury risk in applied settings [22]. Taken together, the small effect of SMI and the absence of a robust TWL signal support the view that subsequent injuries emerge from complex, multifactorial interactions rather than from any single marker [23]. One plausible interpretation is that SMI may partly reflect habitual training exposure and longer-term sport participation, i.e., students with higher muscle mass may have greater exposure to potentially injurious situations which could explain the direction of association despite its very small magnitude; this is consistent with evidence linking skeletal muscle mass indices with physical activity history in university students [24].

When interpreting training-related findings, the operationalization of exposure is important. Accordingly, findings related to training characteristics should be interpreted with caution, as the applied measures capture simplified and partial aspects of training exposure rather than its full physiological and temporal complexity. In our study, TWL was a pragmatic time-based proxy that may not capture intensity distribution, activity type, recovery, or temporal load fluctuations that influence adaptation. Contemporary conceptual models emphasize that training load alone is insufficient to explain injury occurrence and must be understood within broader interactions between intrinsic capacity, exposure, and recovery processes [23]. In this context, injury risk is often framed as a function of how quickly and how far training loads are progressed relative to an individual’s capacity, rather than as a simple consequence of absolute weekly volume alone [25]. Related work suggests that acute: chronic workload dynamics and short-term workload “spikes” (including EWMA-based approaches) may provide additional insight beyond simple weekly totals [26]. These considerations likely contribute to the weak and imprecise association observed for TWL and help explain why combining TWL with SMI did not yield meaningfully stronger discrimination.

A further explanation for the weak associations is that the evaluated variables are relatively distal proxies of injury mechanisms. Body composition indices (FMI/SMI) may reflect long-term morphology and exposure, whereas subsequent injury occurrence is also driven by short-term fluctuations in tissue capacity, recovery, and task-specific loading that were not captured by our measures. In addition, physical activity metrics derived from IPAQ (TPA and VPA%) quantify overall activity and relative intensity distribution rather than the mechanical characteristics of exposure (e.g., impact, direction changes, contact), which may attenuate associations with musculoskeletal injury outcomes.

The absence of a cumulative or interaction effect in our analyses indicates that the combined models did not improve discriminatory performance compared with single markers, suggesting no meaningful synergy between the tested intrinsic and extrinsic variables within this relatively homogeneous, highly active student cohort. Although some studies in other contexts have reported incremental gains when combining indicators (e.g., selected asymmetry-related measures with body composition indices) [27], broader evaluations of musculoskeletal injury prediction modeling show that, even when multiple candidate variables are combined, model performance is often modest, with limited generalizability and practical utility [28]. Evidence on asymmetry-related risk factors is also mixed overall, with inconsistent associations across sporting populations [29]. Importantly, reviews of machine-learning approaches in sports injury research highlight that even advanced analytical methods often achieve only modest and poorly generalizable discrimination when based on limited sets of isolated predictors, supporting the need for broader, multidimensional monitoring frameworks [30]. Collectively, these observations align with our results and reinforce the central practical conclusion that simple anthropometric/body-composition markers and time-based TWL summaries are not sufficient for useful discrimination of subsequent injury status in this population.

The weak discriminatory performance of our models (AUC ≈ 0.60) has clear practical and clinical implications. In applied sports medicine contexts, AUC values below approximately 0.70 are generally considered to reflect poor or only minimal discriminative capability, indicating insufficient accuracy for reliable individual-level injury risk prediction. Consequently, such models offer limited clinical utility for distinguishing between individuals who will and will not sustain a subsequent musculoskeletal injury. From a practical perspective, this suggests that coaches, physiotherapists, and physical education educators should not treat skeletal muscle index (SMI) or training weekly load (TWL) as standalone or clinically actionable prediction tools for subsequent injury risk in previously injured university students. Instead, our findings reinforce the broader conclusion that injury prevention strategies in highly active populations should not be built around simple anthropometric markers or single-volume workload summaries. In the context of this manuscript, ROC findings are interpreted as discrimination/classification within the observed sample, rather than as prospective, time-ordered prediction. Importantly, existing evidence in sports injury prediction research indicates that even increasingly sophisticated analytical approaches often struggle to achieve strong and clinically meaningful discrimination when relying on limited sets of isolated predictors, underscoring the need for broader, multidimensional monitoring frameworks [30]. From an applied standpoint, prevention efforts in university settings are likely better served by focusing on controllable and modifiable domains such as training intensity regulation and progression, movement quality and technical execution, and structured recovery management, while also accounting for psychological and academic stressors. Building on this practical perspective, day-to-day prevention in university settings may be better served by individualized return-to-activity progression after an initial injury and cautious load progression in students with a recent injury history [31]. Routine monitoring of subjective fatigue and early warning symptoms (e.g., soreness, perceived exertion, sleep quality, and overall wellness) may provide more actionable information than weekly load summaries alone, as supported by evidence on single-item athlete wellbeing measures and their relationship with training load [32]. Preventive practice is also facilitated by instructors and coaches who can support implementation and adherence; evidence indicates that coach education can improve adherence to injury-prevention programs in real-world settings [33].

Several limitations of the present study should be acknowledged when interpreting the findings. Due to the cross-sectional design and retrospective assessment of injury history, causal relationships and precise temporal sequencing of injuries cannot be inferred. Training weekly load (TWL) was operationalized as a simple time-based proxy derived from self-reported training frequency and session duration and therefore did not capture dimensions such as exercise intensity, activity type, or progressive overload. Furthermore, because all participants were recruited from sport- and health-related university programs and exhibited generally high physical activity levels, the generalizability of the findings to less active or non-sport student populations may be limited. Vigorous physical activity was expressed as a percentage of total physical activity (VPA%), reflecting relative intensity distribution rather than absolute volume; therefore, this measure does not capture the total amount of vigorous activity performed. Training-related findings should be interpreted cautiously in light of these methodological limitations. Future work would benefit from longitudinal designs, sex- and age-stratified analyses, and broader sets of measured determinants to clarify whether injury-relevant profiles differ across subgroups and exposure environments [14,34,35,36,37]. At the same time, while movement-screening constructs (e.g., Functional Movement Screen), posture-related characteristics, and performance attributes have been examined in the wider literature [38,39,40,41,42], these domains were not assessed in the present study and therefore cannot be evaluated here.

## 5. Conclusions

This study demonstrates that skeletal muscle mass index and weekly training load, whether considered individually or in combination, have limited clinical utility for predicting subsequent musculoskeletal injuries in physically active university students. Although SMI showed a statistically significant association with injury risk, its predictive value was modest and insufficient for reliable individual-level risk stratification. These findings highlight the limited applicability of simple anthropometric and training-volume measures as standalone screening tools. Injury prevention strategies in this population should therefore adopt a multifactorial approach that incorporates biomechanical, behavioral, and recovery-related factors. Future studies should prioritize longitudinal designs to better capture the dynamic mechanisms underlying injury risk in young active adults.

## Figures and Tables

**Table 1:** Participant baseline characteristics (N = 418).

Variable			Males n = 199			Females n = 219			
	Mean	95%CI Lower	95%CI Upper	SD	Mean	95%CI Lower	95%CI Upper	SD	*p*
Age [y]	20.73	20.61	20.85	0.85	20.56	20.46	20.65	0.74	**0.025**
Body height [cm]	182.19	181.20	183.18	7.10	168.17	167.37	168.97	6.01	**<0.001**
Body weight [kg]	79.63	78.25	81.01	9.87	60.86	59.65	62.06	9.05	**<0.001**
BMI [kg/m^2^]	23.97	23.62	24.32	2.48	21.49	21.12	21.85	2.71	**<0.001**
FMI [kg/m^2^]	3.92	3.72	4.12	1.40	5.09	4.85	5.33	1.82	**<0.001**
SMI [kg/m^2^]	17.02	16.60	17.43	2.96	12.74	12.28	13.21	3.48	**<0.001**
TPA [MET/min/week]	3608.2	3418.5	3797.9	1356.9	3019.8	2886.5	3153.1	1000.9	**<0.001**
VPA [%]	22.76	20.93	24.59	13.11	23.95	22.30	25.60	12.40	0.340
TWL [h]	6.14	5.60	6.68	3.88	5.66	5.15	6.17	3.82	0.200
EXP [y]	3.61	3.43	3.79	1.29	3.11	2.92	3.31	1.47	**<0.001**

Abbreviations: BMI, body mass index; FMI, fat mass index; SMI, skeletal muscle mass index; TPA—total physical activity calculated based on the International Physical Activity Questionnaire (IPAQ); VPA—vigorous-intensity physical activity as assessed by the IPAQ intensity component; TWL—weekly training load; EXP—training experience expressed in years; SD—standard deviation; CI—confidence interval. Statistically significant values are highlighted in bold.

**Table 2:** Frequency distributions of injury categories by sex (males: n = 199; females: n = 219).

Injury Category	Sex	0 (n, %)	1 (n, %)	Total (Sex-Specific n)	χ^2^ (df = 1)	*p*
Any injury	males	86 (43.2%)	113 (56.8%)	199	**4.35**	**0.037**
	females	117 (53.4%)	102 (46.6%)	219		
Subsequent injury	males	124 (62.3%)	75 (37.7%)	199	1.27	0.259
	females	148 (67.6%)	71 (32.4%)	219		
Recurrent injury	males	153 (76.9%)	46 (23.1%)	199	0.00	0.998
	females	184 (84.0%)	35 (16.0%)	219		
Other subsequent injury	males	131 (65.8%)	68 (34.2%)	199	1.18	0.277
	females	155 (70.8%)	64 (29.2%)	219		

Note: “1” refers to the presence of the injury category; “0” indicates its absence. For analytical purposes, subsequent injuries were operationalized at different levels. “Subsequent injury” referred to the occurrence of any injury following a previous injury, regardless of anatomical location or injury type. “Recurrent injury” was defined as an injury occurring at the same anatomical location and of the same type as a prior injury. Due to the limited number of cases, injuries classified as related or unrelated were combined into a single category labeled “other subsequent injury” in the statistical analyses. The “Total” column indicates the total number of participants within each sex group and does not represent the overall sample size. Statistically significant values are highlighted in bold.

**Table 3:** Univariate logistic regression models predicting subsequent injury (N = 418).

Predictor	OR	95% CI	*p*-Value	Cohen’s d
Male sex	1.26	0.84–1.89	0.260	0.13
FMI [kg/m^2^]	1.03	0.92–1.16	0.584	0.02
SMI [kg/m^2^]	1.09	1.03–1.15	**0.002**	0.05
TPA [MET/min/week]	1.00	1.00–1.00 *	0.298	~0.00
VPA [%]	1.00	0.98–1.01	0.748	~0.00
TWL [h]	1.03	0.98–1.08	0.307	0.02
EXP [y]	1.03	0.89–1.18	0.716	0.02

Abbreviations: FMI, fat mass index; SMI, skeletal muscle mass index; TPA—total physical activity calculated based on the International Physical Activity Questionnaire (IPAQ); VPA—vigorous-intensity physical activity as assessed by the IPAQ intensity component; TWL—weekly training load; EXP—training experience expressed in years; OR—odds ratio; CI—confidence interval. Statistically significant values are highlighted in bold. * due to rounding, some confidence intervals appear as 1.00–1.00.

**Table 4:** Multiple logistic regression model with interaction term (SMI × TWL) predicting subsequent injury (N = 418).

Predictor	OR	95% CI	*p*-Value	Cohen’s d
SMI	1.07	0.98–1.18	0.139	0.04
TWL	0.98	0.78–1.23	0.833	−0.02
TWL × SMI	1.00	0.99–1.02	0.743	0.001

Abbreviations: SMI, skeletal muscle mass index; TWL—weekly training load; OR—odds ratio; CI—confidence interval.

**Table 5:** Area under the curve (AUC; 95% CI; *p*-values), Youden index, sensitivity, specificity, cut-off values, and AIC for each model, based on the optimal set of injury predictors.

Variable	AUC 95%CI	*p*	Youden	SE	SP	Cutoff	AIC
Combination SMI-TWL	0.607 0.550–0.664	**<0.001**	0.216	0.712	0.504	0.35	536.4
SMI	0.604 0.548–0.661	**<0.001**	0.192	0.685	0.507	14.90	534.7
TWL	0.516 0.457–0.576	0.596	0.057	0.281	0.776	9.0	543.9

Abbreviations: AUC—area under curve, SE—sensitivity, SP—specificity, Cutoff—threshold, AIC—Akaike information criterion. SMI—skeletal muscle mass index; TWL—training weekly load. Statistically significant values are highlighted in bold.

## Data Availability

The data supporting the findings of this study are available from the corresponding author upon reasonable request.
